# Subclinical Hypothyroidism Presenting as Myxedema Coma: Case Report and Literature Review

**DOI:** 10.7759/cureus.25588

**Published:** 2022-06-01

**Authors:** Tsering Dolkar, Michlene Zouetr, Malavika Shankar, Aditya Keerthi Rayapureddy, Zewge Shiferaw-Deribe

**Affiliations:** 1 Internal Medicine, One Brooklyn Health System Interfaith Medical Center, Brooklyn, USA; 2 Family Medicine, American Institute of Antigua College of Medicine, St John's, ATG; 3 Internal Medicine, One Brooklyn Health System Interfaith Medical Center, New York, USA; 4 Internal Medicine/Endocrinology, One Brooklyn Health System Interfaith Medical Center, Brooklyn, USA

**Keywords:** levothyroxine, malaise, subclinical hypothyroidism, internal medicine, endocrinology, icu, bradycardia, hypothermia, myxedema coma

## Abstract

Myxedema coma is a medical emergency with a high mortality rate. Patients with hypothyroidism may develop myxedema coma if left untreated, although quite rare nowadays owing to regular TSH (thyroid stimulating hormone) monitoring. We present the case of a patient with a known history of subclinical hypothyroidism, defined by normal free T4 (thyroxine) and high TSH, who was found to be in myxedema coma. Clinically, the patient was found to be lethargic, bradycardic, and hypothermic, and in the background of high TSH, myxedema coma was suspected. The patient was admitted to the ICU (Intensive Care Unit) and initially treated with intravenous (IV) hydrocortisone for possible concomitant adrenal insufficiency. This was followed by treatment with IV levothyroxine.

## Introduction

Myxedema coma is a rare, often fatal complication of worsening hypothyroidism seen in patients with prolonged hypothyroidism associated with poor medical compliance or lack of disease recognition [1,2]. An estimate of the annual incidence of myxedema coma in the Western world is 0.22 per 1,000,000, as the exact incidence and prevalence of myxedema coma is unknown [3,4]. It is commonly seen in females and in patients >60 years of age, especially during the winter months [4,5]. According to Fliers et al, there are three key diagnostic components: altered mental status, inability to regulate internal body temperature, and triggering events or illnesses, for example, acute infection, myocardial infarction, cold exposure, surgery, sedative use, and thyroid replacement medication noncompliance [1,2]. Furthermore, although it is named “myxedema coma,” patients do not normally present in a coma [1,2]. Patients can present with varying degrees of mentation such as disorientation, extreme fatigue, confusion, or in rare cases, coma [1]. These patients usually have severe hypothyroidism on blood work and only in rare cases is subclinical hypothyroidism seen in patients with myxedema coma in which the TSH is elevated with a normal serum free T4 and free tri-iodothyronine (T3) level [6]. Subclinical hypothyroidism, itself is only present in 4-10% of adults and most are often asymptomatic [7]. In the last decade, there have only been two other case reports of this condition occurring in patients with subclinical hypothyroidism [6,8]. Thus, we present a 72-year-old female with subclinical hypothyroidism (normal free T4 and elevated TSH) who presented with generalized pain and was found to be in myxedema coma.

## Case presentation

A 72-year-old female with a past medical history of Alzheimer’s dementia, old cerebrovascular accident with bilateral upper limb contractures and aphasia, hypothyroidism on levothyroxine treatment with questionable compliance, atrial fibrillation, and hypertension, presented to the Emergency Department (ED) for generalized pain and lethargy. The patient was brought in by her husband due to perceived pain. As per the husband, the patient was moaning all day, which he perceived was an expression of pain. The patient was unable to gesture to the painful part of her body. In the ED, the patient's vital signs were remarkable for a body temperature of 93°F, heart rate of 58 beats per minute, respiratory rate of 18, and blood pressure of 100/64 mmHg. The patient was nonverbal at baseline and appeared lethargic. However, on physical exam, she responded to painful stimuli. Other notable features on physical exam were torticollis, dry flaky skin, delayed deep tendon reflexes, and a large sacral decubitus ulcer. Laboratory investigations revealed an elevated TSH of 59.9 uIU/ml (0.4-4 uIU/ml) and a normal free T4 of 0.82 ng/dL (0.78-2.19 ng/dL) (Table 1). Furthermore, baseline investigations on admission were done (Table 2).

**Table 1:** TSH and free T4 trend during the course of hospitalization. TSH: thyroid-stimulating hormone; T4: thyroxine; NG: nasogastric.

Time	Thyroid Stimulating Hormone (TSH) (uIU/mL)	Free Thyroxine (T4) (ng/dL)	Heart rate (beats/minute)	Temperature (degree Fahrenheit)	Management
On admission	59.9	0.82	38	90.2 (source: Rectal)	Before starting IV levothyroxine
One week later	35.0	1.11	84	94.4 (source: Rectal)	After loading dose of 100 mcg levothyroxine followed by 88 mcg IV daily
Three weeks later	23	1.24	89	97.6 (source: Axillary)	On 125 mcg of levothyroxine via NG tube

**Table 2:** Laboratory investigations on admission WBC: white blood cells; BUN: blood urea nitrogen;  BNP: B-type natriuretic peptide; COVID-19: Coronavirus disease 2019; PCR: polymerase chain reaction; CRP: C-reactive protein; LDH: lactate dehydrogenase.

Investigation	Value	Reference range
Hemoglobin	8.1	11.0 - 15.0 g/dL
Hematocrit	24.3	35-46 %
WBC	2.6	3.8 - 5.3 10x6/uL
Platelets	141	130 - 400 10x3/uL
Glucose	68	80 - 115 mg/dL
BUN	14.1	9.8 - 20.1 mg/dL
Creatinine	0.75	0.57 - 1.11 mg/dL
Sodium	142	136 - 145 mmol/L
Potassium	4,1	3.5 - 5.1 mmol/L
Chloride	115	98 - 107 mmol/L
Bicarbonate	17	23 - 31 mmol/L
Calcium	8	8.8 - 10.0 mg/dL
Albumin	1.9	3.2 - 4.6 g/dL
Corrected Calcium	9	
Magnesium	1.7	1.6 - 2.6 mg/dL
BNP	40.8	10.0 - 100.0 pg/mL
COVID-19 PCR	Negative	
High sensitivity Troponin I	< 3.5	0.0 - 17.0 ng/L
CRP	8.3	0.50 - 1.00 mg/dL
Ferritin	249	11.10 - 264.00 ng/mL
LDH	334	125 - 220 U/L

**Figure 1:**
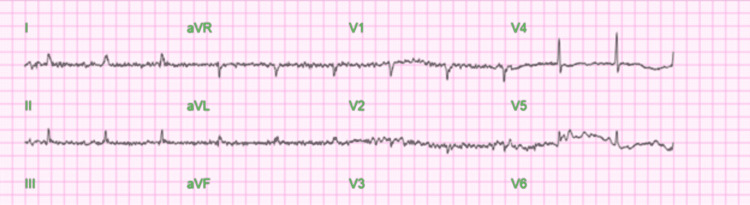
Echocardiogram showed atrial fibrillation with a heart rate of 65 beats per minute, QTc interval was 395 ms and QRS was 88 ms.

**Figure 2:**
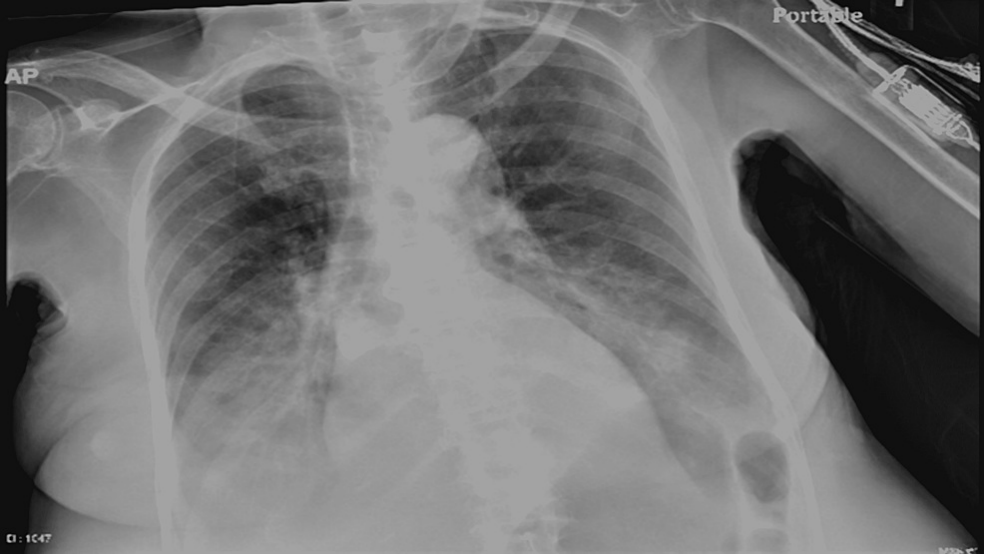
Chest X-Ray: Bibasilar and infrahilar pulmonary opacities concerning for moderate-sized pleural effusions with associated pleural effusion/consolidation secondary to pneumonia and mild pulmonary venous congestion.

Diagnostic studies were obtained. Electrocardiogram (EKG) (Figure 1) showed atrial fibrillation with a heart rate of 65 beats per minute, QT interval corrected for heart rate (QTc interval) was 395 ms and QRS interval was 88 ms. Chest X-ray (Figure 2) revealed bibasilar and infra-hilar pulmonary opacities concerning for moderate-sized pleural effusion with associated pulmonary consolidation, possibly secondary to pneumonia. Computed Tomography (CT) of the chest demonstrated pleural effusion with associated atelectasis and/or consolidation secondary to pneumonia. Echocardiogram showed left ventricular hypertrophy with normal systolic function, an ejection fraction of 55-60%, and mild mitral and tricuspid regurgitation. 

**Figure 3:**
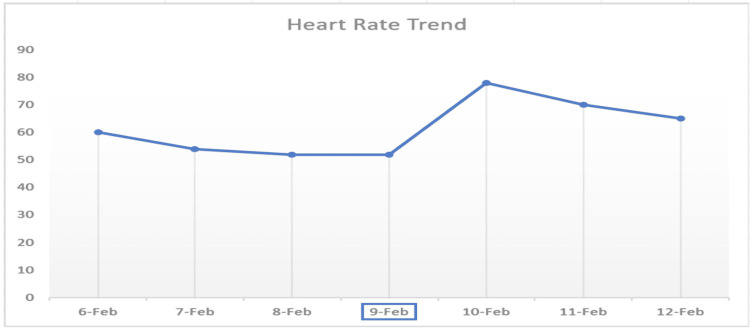
Heart rate trend before and after starting intravenous levothyroxine replacement on 9th February, 2022

**Figure 4:**
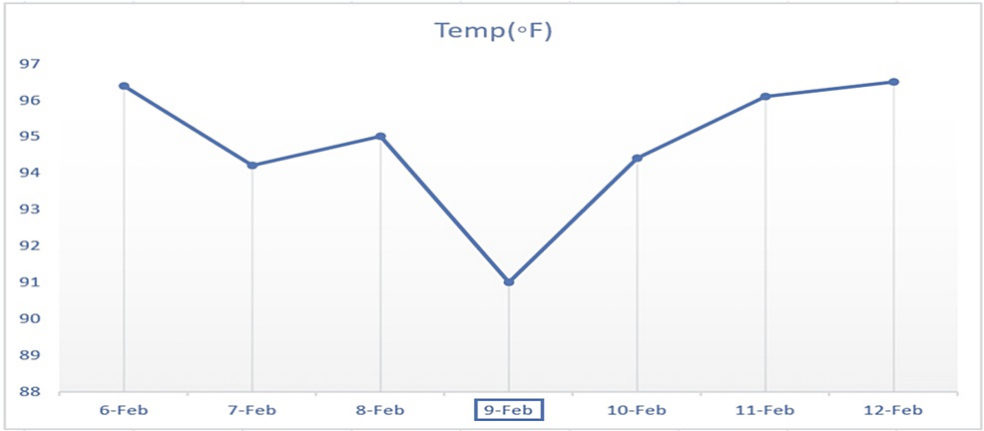
Temperature trend before and after starting intravenous levothyroxine replacement on 9th February, 2022

Owing to the laboratory evidence of subclinical hypothyroidism with physical exam findings of dry/flaky skin, delayed deep tendon reflexes, and vitals suggestive of marked hypothermia with bradycardia, a working diagnosis of myxedema coma was made. The patient was started on hydrocortisone 100 mg IV for possible concomitant adrenal insufficiency as standard practice. The patient was then started on a loading dose of 100 mcg IV of levothyroxine. A lower dose was chosen because of her underlying atrial fibrillation. This was followed by levothyroxine 88 mcg IV daily and tri-iodothyronine 2.5 mcg IV push. Subsequently, the patient was intubated for respiratory failure requiring mechanical ventilation. Additionally, Vancomycin and Meropenem were started for possible sepsis secondary to pneumonia. Thereafter, the patient was upgraded to the ICU for close monitoring. Eventually, over the course of seven days, the patient's temperature improved to 98°F, and her heart rate was consistently above 72 beats per minute. Repeat bloodwork showed improvement in labs (Table 1, Figures 3-4). Also, adrenal insufficiency was ruled out. Hence, tri-iodothyronine 2.5 mcg IV and hydrocortisone were discontinued and levothyroxine 88 mcg IV daily was changed to 125 mcg via nasogastric (NG) tube. Ultrasound of the thyroid could not be done because of the trach collar. Three weeks after admission, a significant improvement was noted. TSH trended down to 23 uIU/mL and free T4 rose to 1.24 ng/dL (Table 1). 

## Discussion

Myxedema coma refers to a condition in which low thyroid hormone results in decreased activity of many organ functions including cardiovascular and mental functions [2]. Mental status changes can manifest as disorientation, extreme fatigue, confusion, or in rare cases, coma [1]. Furthermore, hypothermia can also develop owing to low T3, which has a role in the induction/activation of uncoupling proteins (brown fat) in the mitochondria. The uncoupling of adenosine triphosphate (ATP)-free energy is necessary to increase free energy and in turn, raise core body temperature [9]. Thus, low T3 leads to the failure of both brown-fat metabolic activity and the sympathetic nervous system compensatory activity of vasoconstriction [9]. 

Free T4 (FT4) level may be affected by other factors, such as heparin use and circulating antibodies [10, 11]. This patient was not on heparin when the initial test was done, hence spurious elevation of FT4 was ruled out. Other interfering antibodies or substances are expected to falsely elevate FT4 [11], in which case, the equilibrium dialysis method is preferred for the measurement of FT4. This is a technique that is accessible in bigger clinical research facilities that ultrafilters the undiluted sera to give 36 proper FT4 values in patients with significant modifications to serum T4 binding, including serious nonthyroidal diseases [12]. The FT4 was normal, hence the dialysis method was not required. The dialysis method is used when FT4 is high (above normal) and with clinical discrepancy. In our patient, the FT4 immunoassay was normal. We believe because of acute illness, deiodinase 2 activity was increased on top of already low normal FT4 and consequently, sufficient T3 was not generated. The normal free T4 and high TSH at the admission is by definition subclinical hypothyroidism state, or mild hypothyroidism, even though the patient had a history of hypothyroidism.

Oftentimes, it is difficult to diagnose as the presentation resembles other serious life-threatening conditions such as decompensated heart failure and respiratory failure [13]. However, diagnostic clues arise from overt signs of hypothyroidism such as dry, cold skin, delayed deep tendon reflexes, and coarse hair [13]. Furthermore, based on the original article done by Popoveniuc G et al, a diagnostic scoring system was produced which can indicate a high likelihood of myxedema coma based on clinical signs, symptoms, and causes as seen in Table 3 [14]. In this patient with a past medical history of subclinical hypothyroidism, an acute infection appears to have been the precipitating factor leading to hypothermia, bradycardia, lethargy, bilateral pleural effusions, and obtundation requiring intubation, commonly seen in myxedema coma. In all, our patient had a diagnostic score of 60, according to Table 3, which is highly suggestive of myxedema coma. Patients with a high suspicion of myxedema coma should be treated immediately. Delays especially in laboratory results are fatal and treatment has rarely been shown to be unsafe [2]. Thus, it is important to recognize the clinical presentation.

**Table 3:** Diagnostic Scoring System for Myxedema Coma Adapted from Popoveniuc et al. [14]. **Other EKG changes: QT prolongation, or low voltage complex, or bundle branch blocks, or non-specific ST-T changes, or heart blocks. Total score: >60 - highly suggestive/diagnostic of myxedema coma; 25-59 - supportive of a diagnosis of myxedema coma; <25 - myxedema coma unlikely. GFR: glomerular filtration rate

Thermoregulatory dysfunction (Temperature °F/°C)	Points
>95/35	0
89.6-95/32-35	10
<89.6/32	20
Central Nervous System Effects	
Absent	0
Somnolent/Lethargy	10
Obtunded	15
Stupor	20
Coma/seizures	30
Gastrointestinal Findings	5
Anorexia/abdominal pain/constipation	15
Decreased intestinal motility	20
Paralytic ileus	
Precipitating Event	
Absent	0
Present	10
Cardiovascular Dysfunction	
Bradycardia/Heart rate	
Absent	0
50-59	10
40-49	20
<40	30
Other EKG changes**	10
Pericardial/pleural effusion	10
Pulmonary edema	15
Cardiomegaly	15
Hypotension	20
Metabolic Disturbances	
Hyponatremia	10
Hypoglycemia	10
Hypoxemia	10
Hypercarbia	10
Decrease in GFR	10

Management is initiated with admission to the ICU followed by treatment of the precipitating factor, supportive care of the cardiovascular, respiratory, and nervous systems, and administration of thyroid hormone and glucocorticoids to cover for any coexisting adrenal insufficiency [13]. Hydrocortisone between 50 and 100 mg every 12 hours can be used until adrenal insufficiency is ruled out or a single dose of 2 mg dexamethasone can be given first to start a 1-hour cosyntropin stimulation test to rule out adrenal insufficiency [2]. It is unclear if myxedema coma patients should be treated with T3, T4, or both [13]. According to the American Thyroid Association task force on thyroid hormone replacement, 200 to 400 mcg of IV levothyroxine should be started as a loading dose [13,15]. After which, daily doses of about 1.6 mcg/kg body weight are started with a reduction to 75% if given intravenously [15]. The transition to oral supplementation can only be initiated after the restoration of gastrointestinal function in a clinically improving patient because there is a risk of impaired thyroid hormone absorption and pulmonary aspiration [16]. This was evident in a patient with myxedema coma who was improving on IV thyroid hormone replacement but worsened when transitioned to oral thyroid hormone because of gastric atony [7]. 

Furthermore, a loading dose of 5 to 20 mcg of intravenous tri-iodothyronine is also recommended as a combination with levothyroxine since conversion of thyroxine to tri-iodothyronine may be impaired in myxedema coma [15]. This loading dose is then followed by 2.5 to 10 mcg of intravenous tri-iodothyronine every 8 hours which are to be continued until the patient is clinically improving. Lower doses of thyroxine and tri-iodothyronine should be given to patients with a history of arrhythmia or coronary artery disease as well as elderly and smaller patients [15]. Furthermore, in terms of supportive respiratory care, pulmonary intubation or respiratory assistance should be initiated as hypercapnia and hypoxia can lead to decreased central respiratory drive, alveolar hypoventilation, and altered mental status [13]. Hypothermia, on the other hand, is managed with ordinary blankets since warming blankets can cause vasodilation and shock, especially in patients who have had insufficient fluid resuscitation [1,13]. Furthermore, if there is even the slightest possibility that a bacterial infection is present, antibiotic therapy should be administered [2]. 

Two previous articles have described the presence of myxedema coma in subclinical hypothyroidism. In the first article, a 47-year-old female with a chief complaint of fatigue was found to have subclinical hypothyroidism that led to hospitalization for myxedema coma after poor medication compliance. The patient was treated with IV T3 and experienced drastic improvement [6]. In another article, a patient was admitted for possible nadolol toxicity vs myxedema coma induced by subclinical hypothyroidism, as both present similarly. However, the patient’s symptoms did recur 24 hours after the last dose of nadolol and was resolved with levothyroxine and an epinephrine infusion [8]. In these cases and this patient, the clinical presentation was severe even though thyroid function tests were moderately abnormal.

## Conclusions

It is important to recognize the clinical presentation of myxedema coma and initiate prompt treatment to reduce mortality in patients with a high clinical suspicion despite slightly deranged labs seen in subclinical hypothyroidism or in mild hypothyroidism.
